# Laparoscopic upper pole partial splenectomy for a complex splenic cyst with selective clamping of the magistral blood supply—a case report

**DOI:** 10.1093/jscr/rjaf183

**Published:** 2025-03-20

**Authors:** Jessica L O’Sullivan, Patrick Walker, Suresh Navadgi

**Affiliations:** Department of General Surgery, Royal Perth Hospital, Wellington Street, Perth 6000, Australia; Department of General Surgery, Royal Perth Hospital, Wellington Street, Perth 6000, Australia; Department of General Surgery, Royal Perth Hospital, Wellington Street, Perth 6000, Australia

**Keywords:** splenectomy, partial, laparoscopic

## Abstract

Splenic resection for splenic lesions creates a particular challenge in the case of splenic preservation. The lack of a standardized approach forces consideration of the anatomy of the lesion and the vasculature of the spleen to ensure safety when performing a laparoscopic partial splenectomy. Below, we present the case of a 34-year-old woman who presented to the emergency department with upper abdominal pain.

## Introduction

Splenic lesions are an uncommon indication for resection. These lesions are more frequently benign. However, in the setting of symptomatic lesions, or the need for definitive biopsy- splenectomy is indicated.

Much is known about the risks of a total splenectomy, including overwhelming post splenectomy infection. In highly selective benign lesions, partial splenectomy offers the potential benefit of splenic preservation [1].

There is debate regarding the optimal amount of remnant spleen that should remain. The consensus in the literature is to preserve 25% of the spleen in order to maintain immunological function [2]. This claim has little supporting evidence in the wider literature.

Laparoscopic splenectomy was first performed by Poulin *et al.* [3]. The success of this technique led to surgeons performing splenectomy in elective resections. Previous studies have demonstrated the potential benefits of this approach with laparoscopic splenectomy now being widely accepted as the gold standard approach for splenectomy. However, it still represents a surgical challenge [1, 2, 4–6].

Partial splenectomy is a novel procedure with a limited number of reports in the literature. Of these, the more common procedure is resection of the lower pole of the spleen. This is due to the easier control of the vasculature of the lower pole. Resection of upper pole lesions is more technically demanding as it requires both control and selective clamping of the upper pole splenic vasculature and control of the short gastric vessels.

In the following case we present a laparoscopic upper pole resection with a magistral vasculature arrangement. This is an interesting case as we report on the technique of a novel procedure which is gaining increasing application for the management of upper pole lesions which would previously have necessitated total splenectomy.

## Case report

**Figure 1:**
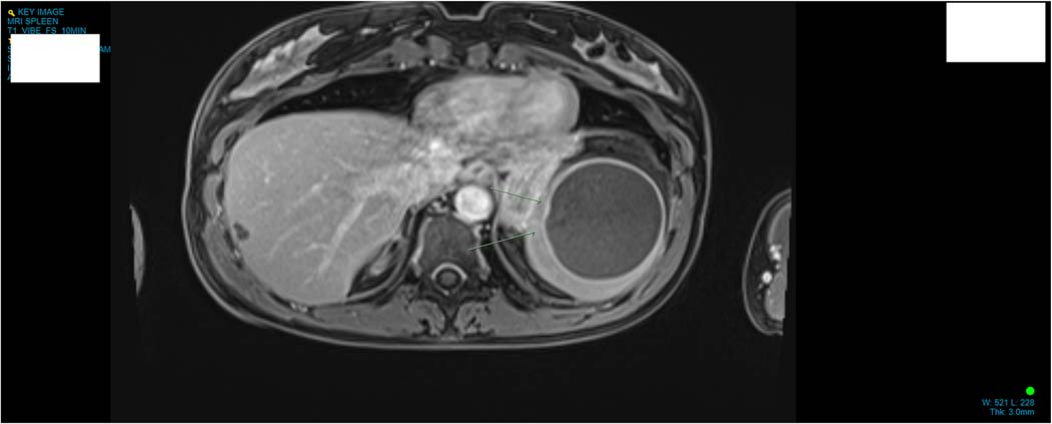
MRI demonstrating anatomical location and size of lesion sagittal plane.

**Figure 2:**
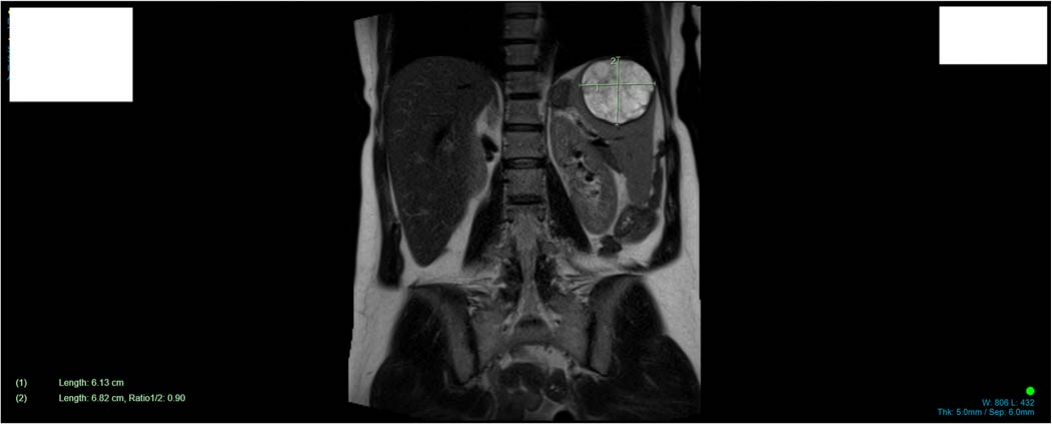
MRI demonstrating size and anatomical location of splenic lesion coronal plane.

A 34-year-old woman presented to the Emergency Department with upper abdominal pain of a few days’ duration with associated nausea. She had no past medical or surgical history. She was hemodynamically stable. Routine blood work and beta human chorionic gonadotrophin were normal. Initial investigation included a Pelvic Ultrasound Scan which showed a complex cystic lesion on the superior aspect of the spleen measuring 64 × 62 × 62 mm. Further investigation with serial imaging including Commuted Tomography (CT), Magnetic Resonated Imaging (MRI) and Proton Emission Topography (PET) demonstrated a cystic lesion of the upper pole of the spleen with an enhancing mural nodule and radiological appearance of a dermoid cyst (Figs 1 and 2). This nodule was found to have low grade avidity on PET scan. The splenic vasculature demonstrated a magistral arrangement on contrast enhanced CT. Hydatid Serology was negative.

Management included annual splenic imaging to monitor for interval increase in size and development of concerning features. Gradually the patient became increasingly symptomatic of the lesion with worsening pain. The decision to proceed to surgery was made.

## Technique

The procedure was carried out under general anesthesia in a reverse Trendelenburg position with the legs in stirrups. The surgeon stands between the patients’ legs. Hasson cannulation is used for entry at the umbilicus. Pneumoperitoneum was established with CO_2_ insufflation pressure of 12 mmHg. One 12 mm port and three 5 mm laparoscopic ports were placed in the upper abdomen under direct vision.

The gastrosplenic ligament was divided with a bipolar energy device with careful ligation of the short gastric vessels (Video 1.) The splenic artery is dissected out and looped proximal to its division into upper and lower pole arteries.

The splenic hilum is carefully dissected to define the vasculature to the upper and lower poles. Vessels to the superior pole of the spleen are ligated and divided. This creates an ischemic line of demarcation within the spleen. The line of transection is marked within the ischemic area (Video 2). Ultrasound is used to confirm adequate surgical margins prior to parenchymal dissection.

Inferior pole vasculature is clamped for total vascular isolation of the spleen. Transection of the splenic parenchyma with the bipolar energy device within the previously marked ischemic area is performed. This is essential to ensure hemostasis. We then divide the remaining splenic ligaments to the upper pole of the spleen. The lower pole clamps are removed at completion. Lastly a fibrin sealant is applied to the cut edge of the spleen to provide ongoing hemostasis. The specimen is removed from the abdominal cavity by an endo-bag through a mini-laparotomy incision at the site of the 12 mm trocar. Pathology of the specimen showed features of a complex pseudocyst without any concerning features for malignancy. All surgical margins were clear. The patient recovered without complication and was discharged home on post-operative day 6. Subsequent blood films did not demonstrate features of asplenia.

## Discussion

The greatest technical challenges in upper pole partial splenectomy are dissection of the hilar vasculature and division of the splenic parenchyma while maintaining effective hemostasis. This includes dissection of the anatomy, proximal control of the splenic vasculature, dissection of the magistral vessels and division of the short gastrics. Division of the splenic parenchyma via the devascularised tissue is critical to minimize blood loss, with special care given to parenchyma within the 1 cm demarcation line. This case presents a complex challenge due to the magistral arrangement of the upper pole vasculature. Knowledge of the anatomy, and appropriate utilization of surgical instruments, energy devices and hemostatic tools to maintain hemostasis is vital.

To conclude, laparoscopic partial splenectomy represents a significant advancement in the management of localized splenic disease, offering patients the potential for improved outcomes. Notably in the case of upper pole lesions careful consideration must be given to the vasculature of the spleen to ensure the safety of the procedure and avoidance of complications.

## Supplementary Material

Short_Gastric_and_Splenic_Short_rjaf183

Transection_small_file_size_rjaf183
